# Predictors of Outcome of Cirrhotic Patients Requiring Invasive Mechanical Ventilation: Experience From a Non-Transplant Tertiary Care Hospital in Pakistan

**DOI:** 10.7759/cureus.21517

**Published:** 2022-01-23

**Authors:** Muhammad Kamran, Abdullah B Khalid, H. A. Basit Siddiqui, Azib Aftab, Rabeea Azmat

**Affiliations:** 1 Gastroenterology, Fazaia Ruth Pfau Medical College, Karachi, PAK; 2 National Institute of Liver and Gastrointestinal Diseases, Dow International Medical College, Karachi, PAK; 3 Nephrology, Karachi Institute of Kidney Disease, Karachi, PAK; 4 Nephrology, The Aga Khan University Hospital, Karachi, PAK

**Keywords:** invasive mechanical ventilation, meld score, ctp score, mortality, cirrhotic patients

## Abstract

Background

Patients with known liver cirrhosis, irrespective of the etiology, have poor outcomes when put on invasive mechanical ventilation in an intensive care unit (ICU) setting. The clinical situation becomes even more complicated when such patients are managed in a non-transplant center. Various factors are associated with poor outcomes, and hence, various scoring systems are available to help determine the prognosis in patients with liver cirrhosis. These scoring systems are broadly classified into two categories, namely, ICU-specific scoring systems and liver disease-specific scoring systems. There is a dearth of data from Pakistan regarding which score better determines the prognosis of patients with liver cirrhosis admitted to the ICU. In this study, we aimed to determine the outcome of cirrhotic patients requiring invasive mechanical ventilation in a non-transplant tertiary care hospital in Pakistan using ICU-specific and liver disease-specific scoring systems.

Methodology

A retrospective study design was applied to a record of 88 cirrhotic patients admitted to the medical ICU of a tertiary care teaching hospital in Karachi, Pakistan, from January 2016 to November 2016. Patients with acute hepatitis were excluded. Data on patients’ characteristics, the reason for intubation, hepatic encephalopathy, the need for vasopressor support, and the duration of ICU and hospital stay were collected. Moreover, the first-day Acute Physiology and Chronic Health Evaluation (APACHE) II, Sequential Organ Failure Assessment (SOFA), Child-Turcotte-Pugh (CTP), and Model for End-Stage Liver Disease (MELD) scores were calculated, with mortality being the primary outcome measure.

Results

The most common etiology was hepatitis C (52.3%, 46/88). The most common reason for intubation was airway protection (57.9%, 51/88). Overall mortality was 71.6% (63/88). On univariate analysis, CTP score >10, MELD score >18, hepatic encephalopathy, bilirubin, prothrombin time, presence of tense ascites, and APACHE II were significantly associated with mortality. On multivariate analysis, CTP score >10 (odd ratio = 21; 95% confidence interval (CI): 4-104; p < 0.001) was an independent predictor of mortality. Area under curve was 0.89 (95% CI = 0.82-0.96) for CTP, 0.86 (95% CI = 0.77-0.95) for MELD, 0.81 (95% CI = 0.69-0.92) for APACHE II, and 0.81 (95% CI = 0.71-0.91) for SOFA in predicting mortality.

Conclusions

CTP and MELD scores are better predictors of short-term mortality in patients with liver cirrhosis requiring invasive mechanical ventilation compared to APACHE II and SOFA scores. CTP score >10 was an independent predictor of mortality.

## Introduction

Pakistan has a huge burden of patients with liver cirrhosis in a resource-constrained setup. Many of these patients experience poor health-related quality of life [1] and a significantly high in-hospital mortality, ranging from 13.5% to 35% [2,3]. Complications associated with liver cirrhosis require liver transplantation; however, this curative modality demands significant resource utilization and is not readily available, even in many tertiary care setups. Patients with liver cirrhosis may require mechanical ventilation for various indications, such as in case of respiratory failure or to protect their airway. Because cirrhotic patients on ventilation often progress to multiorgan failure [4-6], it is debatable to decide whether to intervene aggressively or not because poor prognosis has been associated with mechanical ventilation in this group of patients [7-10].

Although various prognostic scoring systems have been described in the cirrhotic population when admitted to the intensive care unit (ICU) [8,11,12], few studies have reported on the outcome of cirrhotic patients on mechanical ventilation [4,5,13]. It has always been a dilemma as to whether the poor prognosis in such patients is because of the severity of the chronic illness itself or the acute condition with which patients present [14,15]. Similarly, there are conflicting data regarding the effectiveness of ICU-specific scores versus liver-specific scores in predicting the prognosis of patients with liver cirrhosis requiring mechanical ventilation [7,15-18]. The prognosis of critically ill cirrhotic patients on mechanical ventilation is not only determined by the liver disease itself but also depends upon the involvement of other vital organs. Therefore the Acute Physiology and Chronic Health Evaluation (APACHE) II [19] and Sequential Organ Failure Assessment (SOFA) [20,21] scoring systems were developed in studies conducted among the general population [4,7,9,21-23]. These are known as ICU-specific scoring systems and have been shown to outperform liver-specific scoring systems such as the Child-Turcotte-Pugh (CTP) score [21-23]. However, a few recent studies have determined that the Model for End-Stage Liver Disease (MELD) score for assessing the prognosis of cirrhotic patients on mechanical ventilation performs better than the APACHE score [24,25]. Some data also suggest that ICU-specific scoring systems are not reliable enough in predicting the prognosis of cirrhotic patients requiring mechanical ventilation [13].

Newer therapeutic modalities and bridging therapies are easily available options in developed countries for decompensated cirrhotic patients who require mechanical ventilation. However, there remains a huge burden of such patients in developing countries where resources are significantly limited and survival is associated with mechanical ventilation in the absence of liver replacement therapies or liver transplantation. In these settings, the need for utilizing ICU-specific versus liver-specific scoring systems in predicting mortality is a real challenge. This study aims to determine whether ICU-specific scoring systems (APACHE II, SOFA) or liver-specific scoring systems (CTP, MELD) are a better predictor of prognosis in critically ill cirrhotic patients because very limited data are available concerning this aspect of healthcare.

## Materials and methods

**Figure 1:**
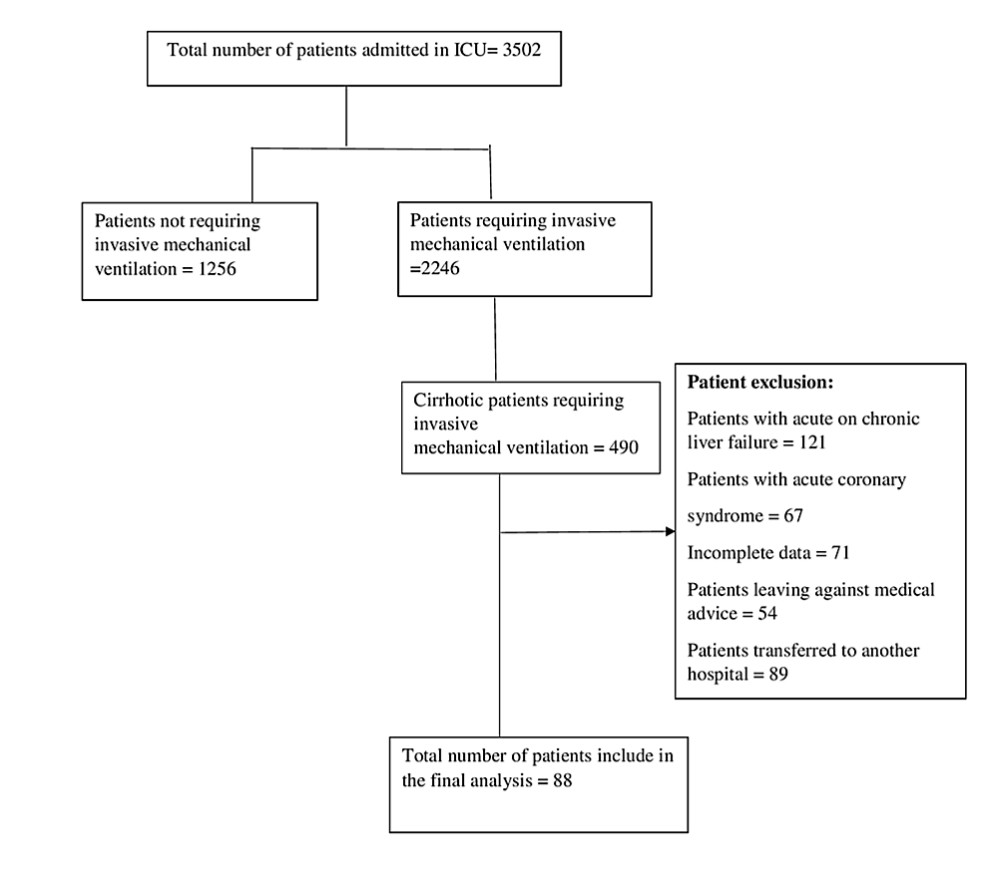
Study flow diagram demonstrating the number of patients identified. ICU: intensive care unit

This descriptive study was conducted in the ICU of Aga Khan University Hospital, Karachi, Pakistan, from January 2016 to November 2016 after obtaining ethical exemption from the Institutional Ethical Review Committee (2521-Med-ERC-13). The study was conducted as per the guidelines of the Declaration of Helsinki. All patients with previously documented liver cirrhosis who underwent mechanical ventilation were included in the study, and the charts of this cohort of ICU patients were extensively reviewed. Patients who fulfilled our inclusion criteria (>18 years of age, a diagnosis of liver cirrhosis based on ultrasonography features and laboratory investigations, and requiring invasive mechanical ventilation as per the discretion of the treating physician) were included. Figure 1 shows the flow diagram of the study.

Data on patients’ characteristics, development of hepatic encephalopathy, need for vasopressor support, and duration of hospital and ICU stay were collected. APACHE II, SOFA, MELD, and CTP scores were calculated on day one of invasive mechanical ventilation (i.e., at baseline), with mortality being the primary outcome measure. The reason for intubation for mechanical ventilation, etiology of cirrhosis, presence of ascites, and spontaneous bacterial peritonitis were also taken into consideration. Presentation with upper gastrointestinal (GI) bleeding, urinary tract infection, pneumonia, hepatorenal syndrome, concomitant hepatocellular carcinoma was also noted, along with the need for hemodialysis and upper GI endoscopy.

Statistical analysis was performed using the Statistical Package for Social Sciences SPSS version 17.0 for Windows (SPSS, Chicago, IL, USA). Categorical variables, such as gender, etiology of liver cirrhosis, presentation with an upper GI bleed, hepatic encephalopathy, urinary tract infection, and aspiration pneumonia, and quantitative variables, such as age, were analyzed. Numeric variables were expressed as mean and standard deviation during the descriptive analysis of the data. A p-value of <0.05 was considered statistically significant. The odds ratio (OR) with a 95% confidence interval (CI) was calculated. Receiver-operating characteristics (ROC) for the different scoring systems in terms of outcome were also calculated.

## Results

We identified 88 patients who satisfied our inclusion criteria. Out of these, only 25 patients were alive at the time of their discharge from ICU, and the remaining 63 died. Table 1 shows the baseline characteristics of the study population. All patients had documented liver cirrhosis based on ultrasonography. The majority of the patients who were put on invasive ventilation had advanced liver disease (CTP class C). The most common reason for intubation was airway protection, followed by severe sepsis, respiratory failure, and cardiac arrest, as shown in Table 2 (57.9%, 51/88). In total, 63 patients died during the hospital course, and 25 patients were discharged in stable condition. Overall mortality was 71.6% (63/88).

**Table 1:** Baseline characteristics of the study population. CTP: Child-Turcotte-Pugh; EGD: esophagogastroduodenoscopy

Characteristics	n	%
Age, in years	50.7 ± 13	
Male	58	66
Female	30	34
Cirrhosis etiology		
Hepatitis B	15	17
Hepatitis C	46	52
Alcohol	11	13
Non-B, non-C	16	18
Ascites		
None	25	28
Mild	36	41
Tense	27	31
Hepatic encephalopathy		
Grade I-II	27	31
Grade III-IV	34	39
Esophageal variceal bleeding	35	40
Hepatorenal syndrome	27	31
Spontaneous bacterial peritonitis	22	25
Urinary tract infection	23	26
Pneumonia	18	21
Concomitant hepatocellular carcinoma	10	11
Need for hemodialysis	10	11
Need for vasopressor support	70	80
CTP class (measured at baseline)		
A	5	5.7
B	26	30
C	57	65
EGD ± intervention during intubation	22	25

**Table 2:** Conditions requiring mechanical ventilation.

Reason for intubation	n	%
Airway protection	51	57.9
Severe sepsis	21	23.9
Respiratory failure	14	15.9
Cardiac arrest	2	2.3

Table 3 shows a comparison of the different continuous variables by patient outcomes. Comparison of various demographic and clinical findings of the study participants by patient outcomes is delineated in Table 4.

**Table 3:** Comparison of continuous variables by patient outcomes. MELD: Model for End-Stage Liver Disease; SD: standard deviation

	Outcome		
Variables	Expired (n = 63)	Discharged (n = 25)	P-value
Age in years mean (SD)	51.1 (13.5)	49.8 (11.9)	0.684
Vitals			
Temperature (°C) mean (SD)	36.8 (0.7)	36.9 (0.6)	0.366
Heart rate (beats/minute) mean (SD)	111.2 (16.1)	104.4 (18.0)	0.088
Respiratory rate (breaths/minute) mean (SD)	20.3 (4.6)	18.8 (4.4)	0.161
Glasgow Coma Scale mean (SD)	8.9 (3.9)	12.2 (3.3)	0.001
Blood count			
Hemoglobin (g/dL) mean (SD)	9.5 (2.5)	8.4 (1.4)	0.039
Platelet’s count (mm^3^) mean (SD)	101.6 (79.3)	109.2 (69.0)	0.679
Biochemical parameters			
Serum bilirubin (mg/dL) mean (SD)	7.4 (8.8)	2.7 (5.4)	0.016
Prothrombin time mean (SD)	25.2 (10.7)	16.0 (5.3)	<0.001
Serum creatinine (mg/dL) mean (SD)	2.3 (1.6)	1.3 (0.9)	0.005
MELD score mean (SD)	27.4 (10.1)	14.9 (7.5)	<0.001
Mean duration on ventilator (days) mean SD)	5.2 (4.1)	3.8 (1.7)	0.113

**Table 4:** Comparison of demographic and clinical findings of the study participants by outcomes. HBV: hepatitis B virus; HCV: hepatitis B virus; NBNC: non-B, non-C hepatitis; CTP: Child-Turcotte-Pugh

		Outcome		Total	P-value
		Expired (n = 63)	Discharged (n = 25)		
Gender	Female	21	9	30	0.5
		(33.3%)	(36.0%)	(34.1%)	
	Male	42	16	58	
		(66.7%)	(64.0%)	(65.9%)	
Cirrhosis etiology	HBV	13	2	15	0.254
		(20.6%)	(8.0%)	(17.0%)	
	HCV	29	17	46	
		(46.0%)	(68.0%)	(52.3%)	
	Alcohol	8	3	11	
		(12.7%)	(12.0%)	(12.5%)	
	NBNC	13	3	16	
		(20.6%)	(12.0%)	(18.2%)	
Ascites	None	14	11	25	0.03
		(22.2%)	(44.0%)	(28.4%)	
	Mild	25	11	36	
		(39.7%)	(44.0%)	(40.9%)	
	Tense	24	3	27	
		(38.1%)	(12.0%)	(30.7%)	
Hepatic encephalopathy	None	10	17	27	<0.001
		(15.9%)	(68.0%)	(30.7%)	
	Grade I-II	23	4	27	
		(36.5%)	(16.0%)	(30.7%)	
	Grade III-IV	30	4	34	
		(47.6%)	(16.0%)	(38.6%)	
Esophageal variceal bleeding		19	16	35	0.004
		(30.2%)	(64.0%)	(39.8%)	
Hepatorenal syndrome		26	1	27	<0.001
		(41.3%)	(4.0%)	(30.7%)	
Spontaneous bacterial peritonitis		20	2	22	0.016
		(31.7%)	(8.0%)	(25.0%)	
Urinary tract infection		17	6	23	0.5
		(27.0%)	(24.0%)	(26.1%)	
Pneumonia		12	6	18	0.401
		(19.0%)	(24.0%)	(20.5%)	
Concomitant hepatocellular carcinoma		8	2	10	
		(12.7%)	(8.0%)	(11.4%)	0.417
Need for vasopressor support		53	17	70	0.084
		(84.1%)	(68.0%)	(79.5%)	
CTP class	A	1	4	5	<0.001
		(1.6%)	(16.0%)	(5.7%)	
	B	10	16	26	
		(15.9%)	(64.0%)	(29.5%)	
	C	52	5	57	
		(82.5%)	(20.0%)	(64.8%)	

**Figure 2:**
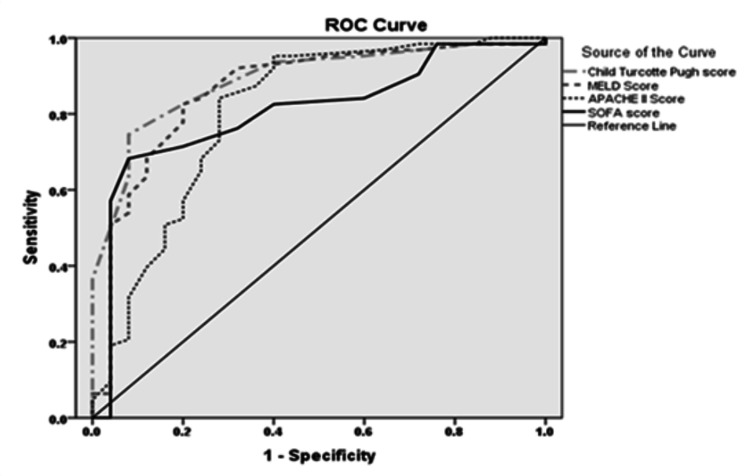
Outcome-oriented ROC of the CTP, MELD, APACHE II, and SOFA scores. ROC: receiver operating characteristics; CTP: Child-Turcotte-Pugh; MELD: Model for End-Stage Liver Disease; APACHE: Acute Physiology and Chronic Health Evaluation; SOFA: Sequential Organ Failure Assessment

According to the univariate analysis, CTP score >10, MELD score >18, hepatic encephalopathy, bilirubin, prothrombin time, presence of tense ascites, and APACHE II were significantly associated with mortality (Table 5). On multivariate analysis, CTP score >10 (OR = 21; 95% CI = 4-104; p < 0.001) was the independent predictor of mortality. P-value was not significant for vasopressor use (p = 0.09) in this population, a finding that was quite astonishing. Area under the curve (AUC) was 0.89 (95% CI = 0.82-0.96) for CTP, 0.86 (95% CI = 0.77-0.95) for MELD, 0.81 (95% CI = 0.69-0.92) for APACHE II, and 0.81 (95% CI = 0.71-0.91) for SOFA in predicting mortality (Figure 2).

**Table 5:** Univariate analysis for determining independent predictors of mortality. CTP: Child-Turcotte-Pugh; MELD: Model for End-Stage Liver Disease; APACHE: Acute Physiology and Chronic Health Evaluation; ICU: intensive care unit; PT: prothrombin time; SBP: spontaneous bacterial peritonitis; OR: odds ratio; CI: confidence interval

Univariate analysis	OR (95% CI)	P-value
CTP score >10	34 (7.15-159.52)	<0.001
MELD >18	17 (5.30-54.46)	<0.001
Hepatic encephalopathy (irrespective of grade)	9.77 (2.61-36.52)	0.001
Need of vasopressor	2.49 (0.84-7.33)	0.09
APACHE II	1.19 (1.09-1.30)	<0.001
Length of ICU stay	0.90 (0.83-0.99)	0.02
Bilirubin (>1.2 mg/dL)	1.17 (1.001-1.38)	0.04
PT (>14 seconds)	1.22 (1.09-1.37)	<0.001
Creatinine (>1 mg/dL)	2.46 (1.23-4.89)	0.01
SBP	5.34 (1.14-25)	0.03
Ascites		
Mild	1.78 (0.61-5.16)	0.28
Tense	6.28 (1.49-26.44)	0.01

## Discussion

Critically ill cirrhotic patients requiring ICU admission and mechanical ventilation have poor overall outcomes [26]. The Cascade of new decompensation, leaky gut leading to bacterial translocation, and ongoing overt hepatic encephalopathy put these patients in the vicious circle of irreversible damage that poses a challenge for intensive care physicians to salvage the disease. Further, although these patients are very sick, most treating clinicians are reluctant to opt for invasive ventilation in this group of patients because facilities for liver transplantation are not readily available everywhere. At the same time, it is very difficult to compare different studies owing to multiple etiologies of liver disease with variable disease severity. Therefore, accurately prognosticating the outcome in patients who need mechanical ventilation for varied indications remains challenging.

The mean age of patients who required ventilatory support in our study was approximately 50 years. This is in contrast to a recently published work from Taiwan, where the mean age was 65 years [27]. The fact that younger cirrhotic patients require ICU care points toward the overall substandard health-related quality of life of such patients in this part of the world. The male predominance noted in our study (66%) was also reflected in data from previous studies [27,28]. With a high prevalence of chronic hepatitis C in Pakistan, this was undoubtedly the most common etiological factor for liver cirrhosis in our study. The mortality was approximately 72% in our cirrhotic patients requiring ICU care and invasive ventilation. Although a very high number, this high mortality rate is comparable to previous studies conducted at reputed centers [29]. A recent study demonstrated lower rates of mortality [24]; however, the number of patients included in the analysis was smaller than that in our study.

Prognostic models have gained considerable significance over the years as they potentially offer an objective assessment of mortality in patients with critical illnesses. Regarding liver cirrhosis and its complications requiring advanced care and mechanical ventilation, predictive models such as CTP, MELD, APACHE II, and SOFA scores have been utilized with variable levels of sensitivity and specificity [13]. According to the univariate analysis, a high CTP score, MELD score, and APACHE II score (signifying advanced liver disease) were significantly associated with increased mortality in extremely sick cirrhotic patients. However, on multivariate analysis, only a high CTP score was an independent predictor of mortality in such patients. The CTP scoring system is a time-tested method for determining the severity of liver disease, as well as for prognosticating mortality among cirrhotic patients [18]. The MELD score, which was initially designed to predict mortality in patients who were candidates for transjugular intrahepatic portosystemic shunt procedure, has also been extensively evaluated as a tool to prioritize patients for liver transplantation, as well as for prognosticating illness severity due to chronic liver disease. However, a recent study showed that the MELD score may not be the best indicator for disease severity in critically ill cirrhotic patients managed in the ICU setting [28,30].

To our knowledge, this study is the first one from this part of the world to objectively analyze the consequences and outcomes in patients with liver cirrhosis placed on invasive mechanical ventilation in a clinical setup where liver transplant facilities are not available. We have shown that such patients have significantly high mortality, and hence, there is a dire need for a well-equipped liver transplant unit in a big metropolitan city like Karachi. However, our study has certain limitations. First, it is difficult to interpret and analyze data presented in a retrospective analysis like ours. However, most of the studies published on this subject in international literature are retrospective in nature with few exceptions [12]. Second, the number of patients included in our study was small. As discussed above, the outcome of patients with end-stage liver disease and requiring mechanical ventilation is generally poor. In our setup, the decision of keeping a patient on mechanical ventilation is based on both the physician’s discretion as well as the family’s consent. Therefore, keeping in view the adverse consequences, in a non-transplant setting, most cirrhotic patients with advanced liver disease end up dying after being categorized as “do not resuscitate.” This phenomenon clearly explains the findings of our study. Third, we could not assess the long-term survival of patients who were successfully weaned off and discharged because many of these patients were lost to follow-up. Lastly, we did not take into account the migration of patients from one CTP class to another during their ICU stay.

## Conclusions

This study showed that in a non-transplant setting, liver-specific scoring systems such as CTP and MELD scores are better predictors of ICU mortality in cirrhotic patients who require intensive care and invasive mechanical ventilation. APACHE II and SOFA scores, although purely designed for the ICU setting, are less sensitive and specific prognostic markers of mortality regarding severely ill patients with cirrhosis in our part of the world. Moreover, we demonstrated a high CTP score (>10) to be an independent predictor of mortality in our patients. There is a high prevalence of liver cirrhosis and its associated complications in a third-world country like ours with limited options for liver transplantation. Therefore, further studies with larger sample sizes are required to prognosticate the disease outcome more scientifically and categorize patients to provide optimum care to such patients.
